# P-1253. Validating and Comparing the Predictiveness of Cefepime Pharmacokinetic Models Used in Bayesian Dosing

**DOI:** 10.1093/ofid/ofae631.1435

**Published:** 2025-01-29

**Authors:** Maria-Stephanie Hughes, Nathaniel J Rhodes, Brandon Smith, Ryan K Shields, Samantha Pan, Erin Weslander, Shannon Galvin, Jasmine Hughes

**Affiliations:** InsightRX, Boston, Massachusetts; Midwestern University, Downers Grove, IL; University of Pittsburgh Medical Center, Pittsburgh, Pennsylvania; University of Pittsburgh, Pittsburgh, Pennsylvania; Northwestern Memorial Hospital, Chicago, Illinois; Northwestern Memorial Hospital, Chicago, Illinois; Northwestern University Feinberg School of Medicine, Chicago, Illinois; InsightRX, Boston, Massachusetts

## Abstract

**Background:**

Bayesian software may enhance beta-lactam dosing. Therefore, we analyzed the predictiveness of cefepime pharmacokinetic (PK) models for use as Bayesian priors.

**Figure:**
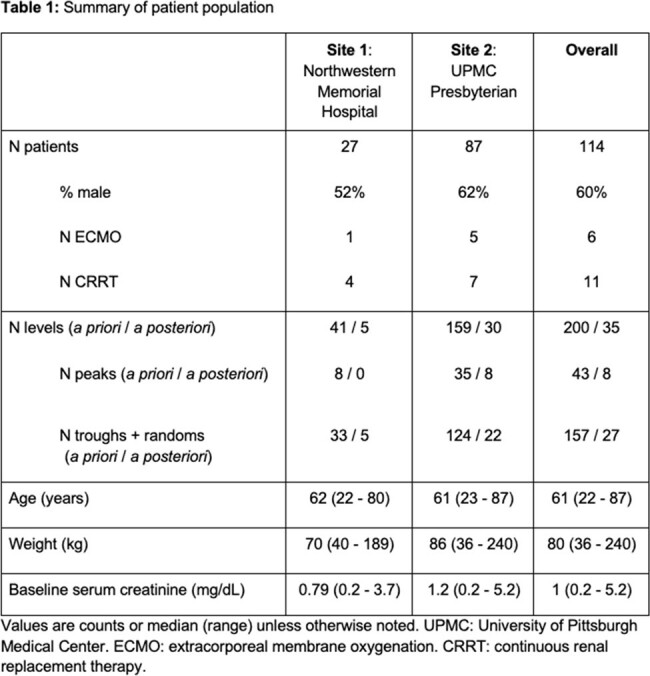

**Methods:**

Data on adult patients receiving cefepime intravenous therapy were entered into a Bayesian software at 2 sites. Inclusion criteria encompassed patients with ≥ 1 cefepime drug concentration (TDM), including those undergoing continuous renal replacement therapy. Exclusion criteria were dialysis and outlier TDMs defined as > 3 standard deviations above the mean. Adult parametric PK models were identified and implemented using PKPDsim. Measured TDMs were predicted iteratively, using model priors to predict the first set (“*a priori*”), with subsequent TDMs predicted using Bayesian estimation (“*a posteriori*”), to mirror the clinical experience (i.e., initial predictions were made using only demographics; updated predictions were made using each additional TDM event). TDMs were analyzed separately as peaks or as random/trough levels. Mean percent error (MPE) assessed bias and root mean square error (RMSE) assessed imprecision. Statistical significance was determined using overlapping 90% confidence intervals from 1000 bootstrap simulations.

**Figure:**
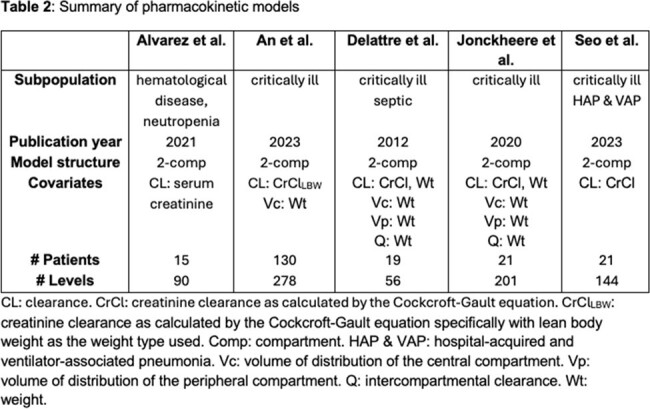

**Results:**

A total of 114 patients with 200 TDMs for *a priori* and 35 TDMs for *a posteriori* predictions were included (Table 1). Five PK models were identified (Table 2). The An et al. and Jonckheere et al. models were most predictive, with acceptable RMSE ≤ 25 mg/L and bias < 25% (Fig 1.). The An et al. model was most precise *a priori* (RMSE_peak_: 23 mg/L, RMSE_random/trough_: 19 mg/L versus other models ranging from 26-33 mg/L and 23-30 mg/L) but demonstrated bias in predicting peaks *a priori* (MPE: 24%) that was not improved *a posteriori*. The Jonckheere et al. model demonstrated similar precision *a priori* to the An et al. model and showed better precision in predicting peaks *a posteriori* (RMSE_peak_: 6 mg/L versus other models ranging from 13-25 mg/ L; Fig 1.).

**Figure:**
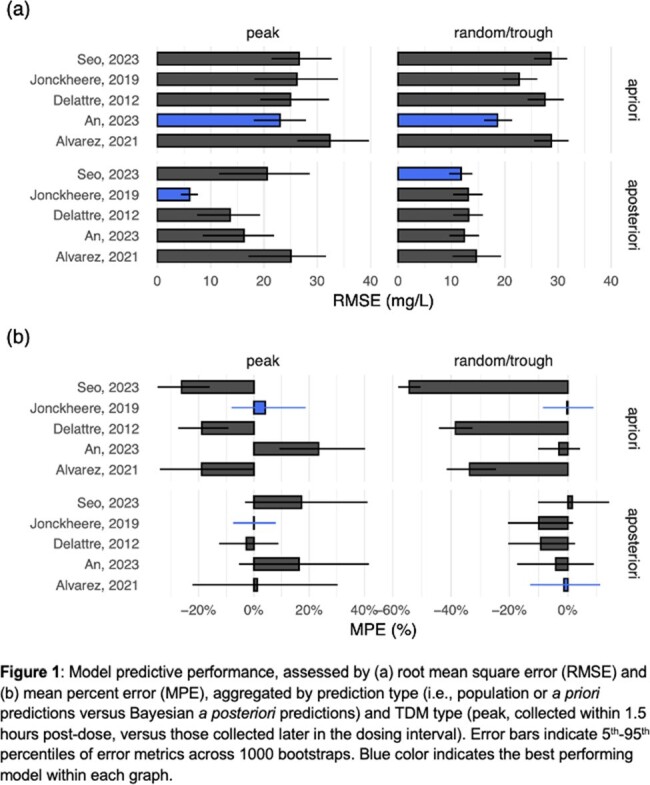

**Conclusion:**

While many models performed similarly (overlapping 90% CI), the An and Jonckheere models both demonstrated acceptable bias and precision for predicting random/trough levels. Centers using a random/trough sampling approach to estimate cefepime exposures may find these models clinically useful.

**Disclosures:**

**Maria-Stephanie Hughes, PharmD**, InsightRX: Employee of company|InsightRX: Stocks/Bonds (Private Company) **Nathaniel J. Rhodes, PharmD MS**, Apothecademy, LLC: Advisor/Consultant **Brandon Smith, MD, PharmD**, Melinta Therapeutics: Advisor/Consultant|Shionogi, INC: Advisor/Consultant **Ryan K. Shields, PharmD, MS**, Allergan: Advisor/Consultant|Cidara: Advisor/Consultant|Entasis: Advisor/Consultant|GSK: Advisor/Consultant|Melinta: Advisor/Consultant|Melinta: Grant/Research Support|Menarini: Advisor/Consultant|Merck: Advisor/Consultant|Merck: Grant/Research Support|Pfizer: Advisor/Consultant|Roche: Grant/Research Support|Shionogi: Advisor/Consultant|Shionogi: Grant/Research Support|Utility: Advisor/Consultant|Venatorx: Advisor/Consultant|Venatorx: Grant/Research Support **Jasmine Hughes, PhD**, InsightRX: Employee|InsightRX: Stocks/Bonds (Private Company)

